# Modified Damus-Kaye-Stansel Anastomosis to Prevent Coronary Obstruction Between the Great Arteries

**DOI:** 10.1016/j.atssr.2024.01.017

**Published:** 2024-02-29

**Authors:** Takashi Nagase, Shinichiro Oda, Yoshinobu Maeda, Jin Ikarashi, Shuhei Fujita, Yasutaka Goto, Masaaki Yamagishi

**Affiliations:** 1 Department of Pediatric Cardiovascular Surgery, Children’s Medical Center, Kyoto Prefectural University of Medicine, Kyoto, Japan

## Abstract

The conventional Damus-Kaye-Stansel procedure may cause coronary artery compression when the coronary arteries are situated between the great arteries. We have performed a modified Damus-Kaye-Stansel procedure utilizing a “flap-bridging technique,” in which an inverted U-shaped flap incised from the aorta is bridged to the main pulmonary trunk, creating sufficient space between the great arteries, in an 8-month-old boy who was a Fontan candidate with congenitally corrected transposition of the great arteries. This modified approach yielded favorable outcomes without coronary events and can effectively prevent coronary obstruction in cases where the coronary arteries run between the great arteries.

Damus-Kaye-Stansel (DKS) anastomosis is an effective procedure for relieving systemic outflow tract obstruction. However, the conventional DKS procedure may cause coronary artery compression in cases where the coronary arteries are positioned between the great arteries. In such cases, a modification of DKS anastomosis is needed.

An 8-month-old male Fontan candidate was diagnosed at birth with congenitally corrected transposition of the great arteries, restrictive ventricular septal defect, parachute mitral valve, severe tricuspid regurgitation, and mild subpulmonary stenosis. At the age of 2 months, he underwent main pulmonary artery banding, tricuspid valve plasty (De Vega), and atrial septal defect creation. He awaited a bidirectional cavopulmonary shunt; however, restrictive ventricular septal defect and subpulmonary stenosis necessitated a simultaneous DKS anastomosis.

Preoperative computed tomography revealed an anterior-posterior relationship between the great arteries (GAs). The aortic and pulmonary commissures were misaligned and both the left and right coronary arteries originated from the facing sinus. The orifices were in close proximity. The right coronary artery ran between the GAs (Figures 1A-1C).

**Figure 1:**
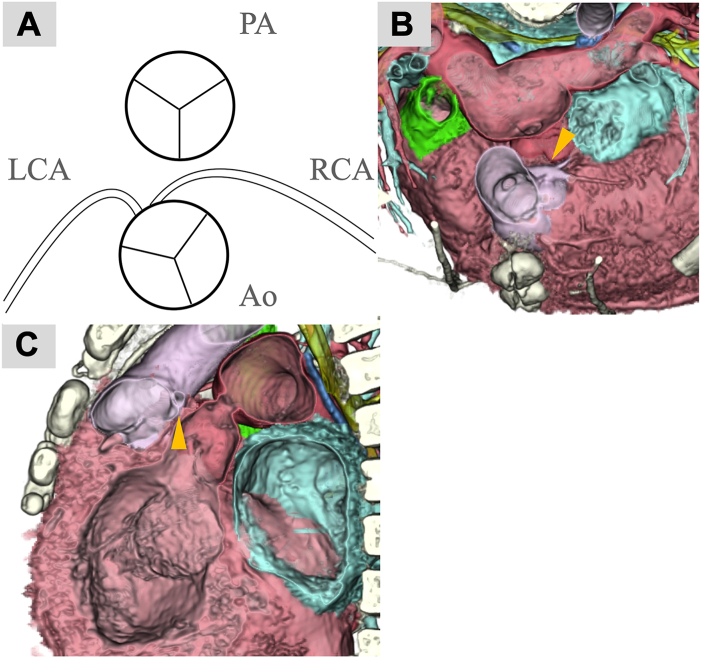
Anatomical relationship between the coronary arteries and great arteries (GAs). The relationship between the GAs is almost anterior-posterior. The aortic and pulmonary commissures are not aligned. Both the left and right coronary arteries originate from the facing sinus. (A) The orifices are in close proximity. (B, C) Right coronary artery (arrowhead) runs between the GAs. (Ao, aorta; LCA, left coronary artery; PA, pulmonary artery; RCA, right coronary artery.)

The surgery was performed through a median resternotomy. After establishing cardiopulmonary bypass in the usual manner, an aortic cross-clamp was placed and the cardioplegic solution was infused. The main pulmonary artery (mPA) was debanded and distally transected as much as possible. An inverted U-shaped incision was made on the posterior wall of the aorta (Figure 2A). The flap created from this U-shaped incision was bridged to the anterior wall of the proximal side of the mPA to ensure adequate space around the coronary arteries (Figure 2B). A patch was created using the pulmonary arterial wall on the peripheral side of the mPA and sutured to cover the bridging flap (Figure 2C). A schematic of this technique is shown in Figure 2D. The site from which the mPA patch was harvested was closed directly. Bidirectional cavopulmonary shunt was performed after declamping the aorta.

**Figure 2:**
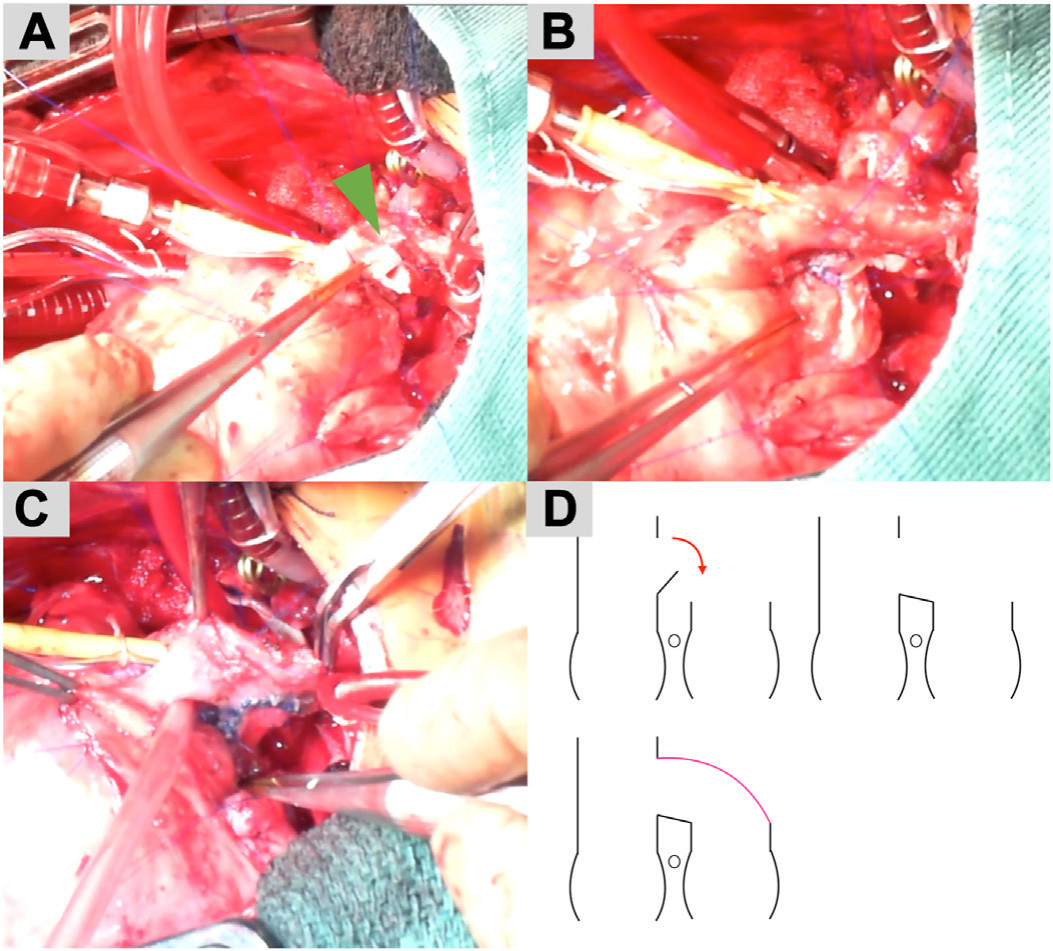
Operative images. (A) An inverted U-shaped incision (green arrow) is made in the posterior wall of the aorta. (B) The flap created from this U-shaped incision is bridged to the anterior wall of the proximal main pulmonary artery (mPA), ensuring adequate space around the coronary arteries. (C) A patch is created using the pulmonary arterial wall on the peripheral side of the mPA and sutured to cover the bridging flap. (D) A schematic of this technique.

No coronary-associated events occurred during the postoperative period, and postoperative electrocardiography revealed no ischemic changes. Postoperative computed tomography revealed no stenosis at the DKS anastomosis site or coronary artery obstruction (Figure 3A). A postoperative catheter examination revealed a smooth DKS pathway (Figure 3B). The patient was discharged 68 days after surgery.

**Figure 3:**
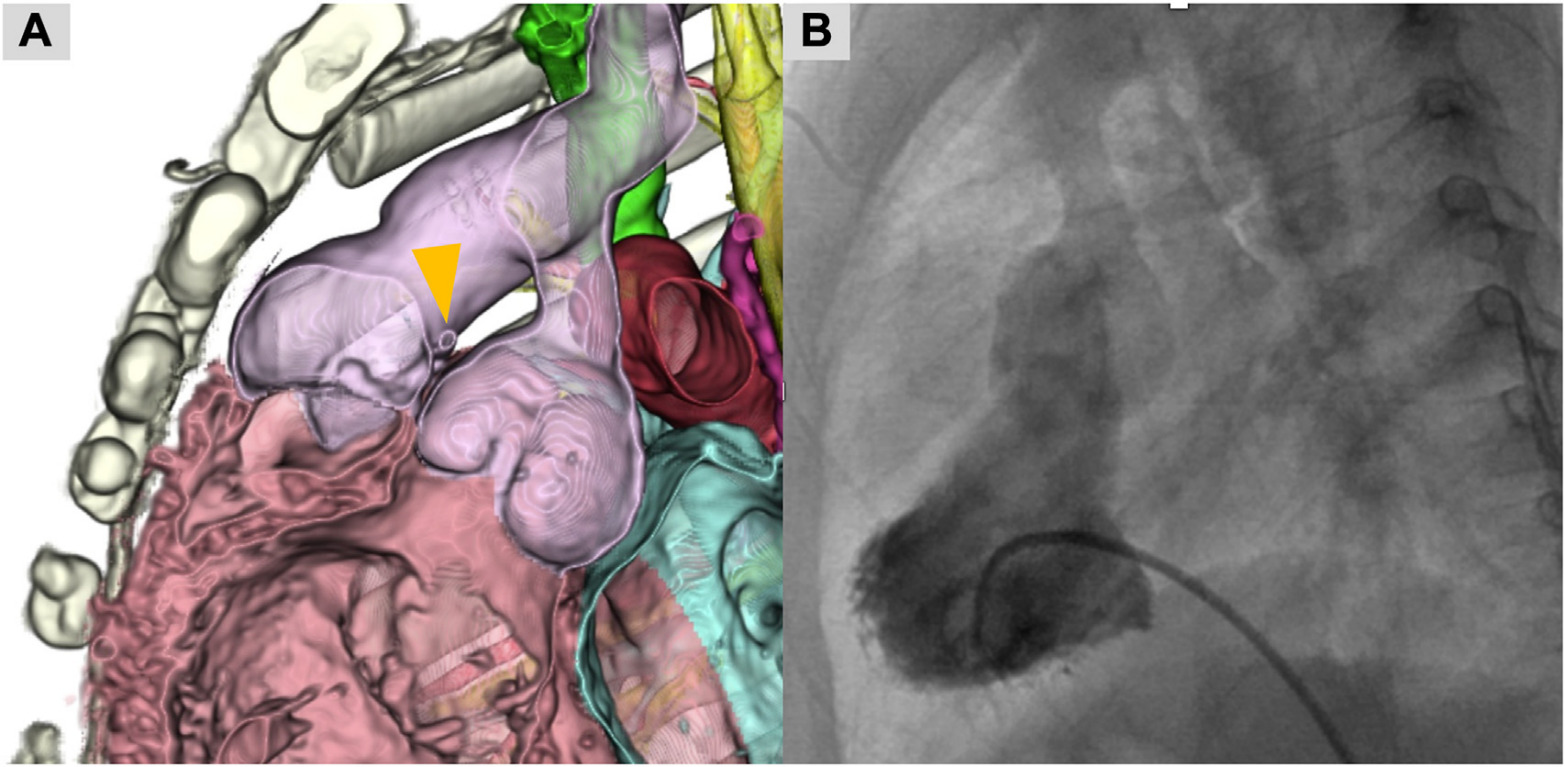
Postoperative computed tomography (CT) and catheter examination. (A) CT shows no stenosis at the Damus-Kaye-Stansel (DKS) anastomosis site (arrowhead) or coronary artery obstruction. (B) A postoperative catheter examination shows a smooth DKS pathway.

## Comment

DKS anastomosis is an effective procedure for relieving systemic outflow tract obstruction, especially in univentricular patients, and is used in various congenital heart diseases.^1^^,^^2^

However, when the coronary arteries are positioned between the GAs, there is a risk of coronary artery obstruction due to compression by the conventional DKS procedure.^3^ Therefore, it is crucial to assess the course of the coronary arteries before proceeding with DKS.

The key points of this technique are the “flap-incision site” and “flap-bridging site.” Care should be taken to avoid incising beneath the sinotubular junction of the aorta. To ensure an appropriate incision site, a U-shaped incision should be made with cardioplegic infusion to avoid collapse of the aorta.

The height of the mPA spontaneously determines flap-bridging height. To ensure sufficient space around the coronary arteries, it is essential to transect the mPA as distally as possible. The pulmonary arterial wall was used to cover the DKS pathway. If an adequately sized pulmonary artery wall cannot be obtained, artificial materials such as an expanded polytetrafluoroethylene graft patch can be used as a substitute. However, this procedure raises concerns regarding the compression of the coronary arteries due to future root dilatation. In such situations, right coronary artery transfer may be necessary. Long-term follow-up is needed to assess the morphologic changes in DKS anastomosis. In conclusion, this modified DKS anastomosis effectively prevents coronary obstruction in cases in which the coronary arteries run between the GAs.
